# Robotic Surgery for Sigmoid Colon Cancer in a Patient With Severe Obesity and Protocol Proposal of Preoperative Weight Reduction Program

**DOI:** 10.7759/cureus.84540

**Published:** 2025-05-21

**Authors:** Nobuko Matsuoka, Yasutake Uchima, Shuhei Ota, Yoshitake Endo, Teruyoshi Amagai

**Affiliations:** 1 Department of Surgery, Yao Tokushukai General Hospital, Osaka, JPN; 2 Department of Surgery, Chubu Tokushukai General Hospital, Okinawa, JPN; 3 Faculty of Health Care Sciences, Department of Clinical Engineering, Jikei University of Health Care Sciences, Osaka, JPN

**Keywords:** colon cancer, corrected body weight, obesity, robotic surgery, weight reduction program

## Abstract

Surgical procedures in patients with severe obesity are associated with an increased risk of complications. Preoperative weight reduction is recommended. We report a case of robotic-assisted sigmoidectomy in a patient with severe obesity (obesity class III: WHO classification) and sigmoid colon cancer after preoperative management based on the previously reported protocol. A 47-year-old man was referred from a private clinic because of an occult fecal blood test during a routine annual check-up. The colonoscopy with pathological examination and contrast-enhanced computed tomography (CE-CT) revealed stage cT3N1bM0 sigmoid colon cancer. Since he was allowed to wait up to six weeks before colon cancer surgery to avoid intraoperative and postoperative complications, we organized the multidisciplinary team for a preoperative multidisciplinary weight reduction program (WRP) based on corrected body weight (cBW), adding physical therapy, smoking and alcohol cessation counseling, sleep apnea screening, and continuous positive airway pressure (CPAP) therapy. He successfully reduced the BW from 122 to 112 kg and underwent robotic surgery for sigmoid colon cancer with a minimal blood loss of 22 mL. Based on our current experience, we propose preoperative WRP using a concept of cBW for super-obese surgical patients whose BMI is above 40 kg/m^2^.

## Introduction

Surgical procedures in patients with severe obesity are associated with increased blood loss, prolonged operative time, a higher rate of conversion to open surgery, and an increased risk of complications. Preoperative weight reduction is therefore recommended [1]. The guidelines recommend a very-low-calorie diet of 400 to 800 calories per day before surgery [2]. However, there is no recommendation for the amount of energy per kilogram of body weight or adjusted body weight (adBW). However, implementing dietary and exercise guidance for weight reduction is often challenging due to the difficulties in obtaining patient understanding, cooperation, and the pathological heterogeneity of their obesity reason. Here, we report a case of robotic-assisted sigmoidectomy in a patient with severe obesity (Obese Class III: WHO classification, or we define this obesity as super-obese in the current report) and sigmoid colon cancer, following preoperative management based on the ERABS (Enhanced Recovery After Bariatric Surgery) protocol [3].

## Case presentation

**Figure 1:**
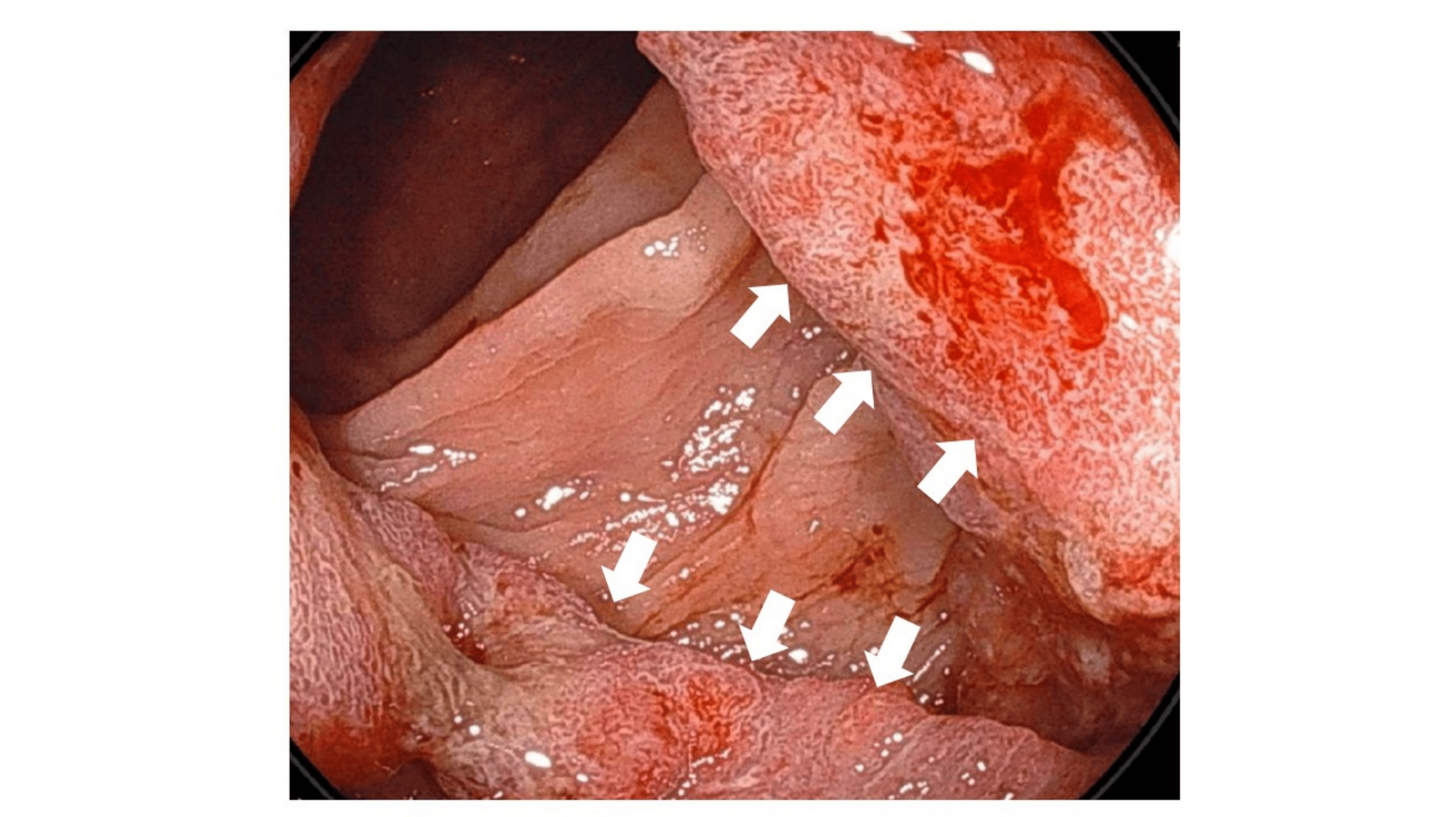
The colonoscopy findings of the sigmoid colon cancer The colonoscopy revealed semicircular bulging, and the biopsy confirmed adenocarcinoma of the sigmoid colon. The arrows in the direction of the right and bottom left show the pathologically diagnosed adenocarcinoma of the sigmoid colon.

A 47-year-old male was referred from a private clinic because of an occult fecal blood test during a routine annual check-up. A colonoscopy performed by an internal medicine physician at our hospital revealed a tumor in the sigmoid colon. His biopsy pathology revealed adenocarcinoma of the sigmoid colon. His smoking history was 20 cigarettes a day for 27 years, and drinking 700 mL of beer a day. The colonoscopy revealed sigmoid colon cancer, which was proven to be adenocarcinoma by pathological examination (Figure 1).

**Figure 2:**
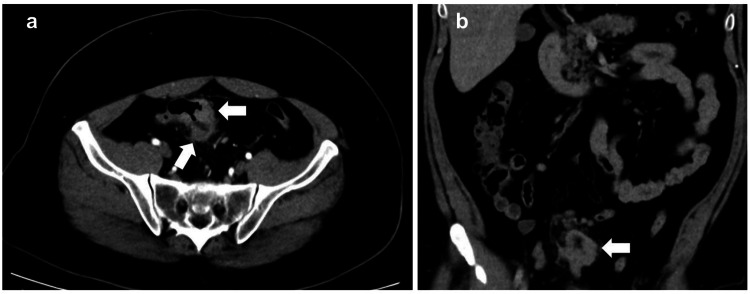
The contrast-enhanced computed tomography (CE-CT) images The arrows of sub-images a and b reveal the sigmoid colon wall thickening, and pathologic biopsy results showed adenocarcinoma. Images a and b shown in cross and coronal sections, respectively.

The contrast-enhanced computed tomography (CE-CT) revealed stage cT3N1bM0 of sigmoid colon. On the same CT images, his abdominal subcutaneous fat depth was 68 mm at 10 mm above the umbilicus of the port insertion point (Figure 2).

At this time, our concern was his severe obesity with a body mass index of 44 kg/m^2^, for height 1.60 m and weight 112 kg. To achieve a meticulous radical sigmoid colectomy with deep lymph node dissection, robot-assisted sigmoidectomy was planned. Since it was allowed to wait up to six weeks until the colon cancer surgery to avoid intraoperative and postoperative complications, we set up the multidisciplinary team for a preoperative multidisciplinary weight reduction program (WRP) as a nutritional intervention. Nutritional intervention was part of the WRP, including daily nutritional requirements consisting of 1,700 kcal of energy and 60 g of protein. adBW information is calculated using the following equation: (Current BW - IBW) × 0.4 + IBW [4], where IBW is the ideal body weight and corresponds to a body weight of BMI =22, because at a BMI of 22, morbidity and mortality are lowest between the ages of 19 and 84 [5]. From this calculation, his adBW was calculated to be 78 kg. As the results were based on adBW, daily energy and protein per adBW were 22 kcal and 0.76 g, respectively. In addition, his lipid profile of dyslipidemia, such as total cholesterol, triglyceride, and LDL-cholesterol, was improved (Table 1).

**Table 1:** The laboratory data before and after weight reduction program ALP: alkaline phosphatase,  ALT: alanine aminotransferase, AST: aspartate aminotransferase; BUN: blood urea nitrogen; CBC: complete blood count; CRP: C-reactive protein; eGFR: estimated glomerular filtration rate; GLU: glucose; HbA1c: hemoglobin A1c;  HDL-C: high-density cholesterol; HGB: hemoglobin; LDL-C: low-density cholesterol;  N: not measured; PLT: platelet; T-BIL: total bilirubin; T-CHO: total cholesterol; TG: triglycerides; WBC: white blood cell.

Categories		Before	After	Unit	Reference
CBC	WBC	121	83	10^2^/μL	33-86
	HGB	14.6	13.3	g/dL	13.7-16.8
	PLT	32	21.2	10^4^/μL	15.8-34.8
Biochemistry	CRP	0.2	0.35	mg/dL	<0.14
	Albumin	4.1	4	g/dL	4.1-5.1
	T-BiLL	0.6	0.7	mg/dL	0.4-1.5
	ALP	112	90	U/L	38-113
	AST	15	17	U/L	13-30
	ALT	19	19	U/L	10-42
	T-CHO	283	192	mg/dL	142-248
	TG	271	112	mg/dL	33-149
	HDL-C	51	50	mg/dL	38-90
	LDL-C	231	142	mg/dL	65-163
	GLU	92	NA	mg/dL	73-109
	BUN	6.2	6.9	mg/dL	8.0-20
	Creatinine	0.76	0.76	mg/dL	0.65-1.07
	eGFR	86.8	74.8	mL/min/1.73 m^2^	≥60
	Na	139	38	mmol/L	138-145
	K	4.6	4.5	mmol/L	3.6-4.8
	Cl	103	104	mmol/L	101-108
	HbA1c (NGSP)	5.80	NM	%	<6.0

Moreover, physical therapy, smoking and alcohol cessation counseling, sleep apnea screening, and continuous positive airway pressure (CPAP) therapy were provided as WRP. This WRP was performed for 30 days with energy and protein administration of 24 kcal and 0.85 g per adBM, respectively. All of these WRPs have been conducted in a hospital setting because early medical intervention may be necessary if any adverse events related to the program occur. As a result of running this WRP, he achieved a preoperative weight reduction from 122 kg to 112 kg, corresponding to a BMI reduction from 44 to 40 kg/m^2^. After perioperative management metrics (PMM) achievement, the robot-assisted sigmoid colectomy was successfully completed. He experienced no pain after the surgery and was able to get out of bed and walk the following day. In addition, there were no obesity-related complications or wound dehiscence. He was discharged home on postoperative day 10.

## Discussion

The importance of preoperative nutritional management for weight reduction

Preoperative nutritional interventions focus on correcting comorbidities and nutritional deficiencies, particularly hypovitaminosis and micronutrient imbalances, through a multidisciplinary program involving nutritionists, physicians, and fitness trainers [6]. A preoperative nutritional management aims to achieve metabolic control and reduce the comorbidities associated with the procedure. Cobalamin (Vitamin B12) deficiency is common, often linked to the use of medications for obesity-related comorbidities, such as metformin, proton pump inhibitors, angiotensin-converting enzyme inhibitors, and colchicine, with small intestinal bacterial overgrowth further exacerbating the condition [7]. Furthermore, triglyceride levels dropped below 150, which is the lower limit of the diagnostic criteria for hyperlipidemia, before and after the intervention. The risk of acute pancreatitis associated with hyperlipidemia was reduced, and the risk of delaying surgery was avoided.

Preoperative management does not change colon cancer outcomes for six weeks

Preoperative caloric restriction has been reported to reduce visceral fat and decrease liver volume by 16-20% [1]. There is a concern that postponing surgery for colorectal cancer due to WRP may lead to progression of the colorectal cancer. As a result of a scientific review of this concern, it has been shown that the waiting time to surgery can be extended by six weeks without worsening the survival of patients with colorectal cancer younger than 50 years [8].

Protocol proposal of nutritional intervention - WRP

When planning gastrointestinal cancer surgery for a critically ill obese patient, as in this case, we recommend the following nutritional weight loss management protocol during the six-week preoperative waiting period, the period during which waiting for surgery does not worsen prognosis. According to our case with preoperative WRP for super-obese patients whose BMI is over 40 kg/m^2^, we would propose the protocol of preoperative nutritional intervention as part of WRP. The details are shown in Table 2. However, this is a preliminary, exploratory protocol requiring further validation in larger cohorts.

**Table 2:** The protocol of nutritional intervention as the preoperative WRP according to obesity severity adBW: adjusted body weight; BMI: body mass index; WRP: weight reduction program.

Category	Degree of severe obesity			Unit
	Mild	Moderate	Severe	
BMI	40-44.9	45-49.9	≥50	kg/m^2^
Nutritional category				
Energy	20	15	10	kcal/adBW/day
Protein	1.0-1.2			g/adBW/day
Fluid	30			mL/adBW/day
Nitrogen balance (NB)	Calculation equation written in the text [9]			g/day

Nitrogen balance (NB) available in nutrition intervention

The equation for calculating nitrogen balance (NB) is as follows: NB = N[IN] - N[OUT] = Protein (g)/6.25 - [UUN (mg/dL) × Urine Volume (dL)] × 10^-3^ × 1.25 [9]. Here, UUN represents urinary urea nitrogen concentration. The NB reflects the stress severity of the patient, and an NB close to 3 g/day reveals being in the stable state. In the situation of decreasing NB, the patient is under stress and is losing muscle, and daily nutrient amounts must not be increased, and vice versa. When signs of adverse events such as liver or kidney damage, dehydration, water intoxication, or refeeding syndrome are suspected during a weight loss program as a nutritional intervention, the program should be immediately discontinued and appropriate medical management initiated.

Limitations of the current reporting

This case report has several limitations, which are described below. First, the outcome of robotic colon cancer surgery without a weight loss program is unclear. In other words, there is no basis for evaluating the effectiveness of such a program. Robotic surgery allows for precise surgical operations. It is believed that the effectiveness of the WRP will become clear when it is compared with a control group. However, it is not practical to perform a surgery without any intervention due to ethical issues. Second, changes in body composition, particularly reductions in fat volume and rate, were not measured before or after the nutritional intervention of the WRP. If these changes could be measured, they would be useful as an index in addition to body weight. Third, other than LDL cholesterol, no effects of WRP intervention have been observed based on blood data. In other words, although dyslipidemia was present, diabetes and renal dysfunction were not. Therefore, it was not possible to clarify the hematological effects before and after the nutritional intervention. It seems necessary to clarify blood data, body composition data, and indicators of the intervention effect within 30 days.

## Conclusions

Robotic surgery is a feasible and safe option for patients with severe obesity, allowing for the successful completion of complex procedures. In addition, preoperative management based on the ERABS protocol enhances surgical safety. The current case of a 47-year-old male has undergone WRP and an uneventful recovery after the operation. We propose a preoperative WRP based on the concept of adBW. However, this is a preliminary, exploratory protocol requiring further validation in larger cohorts.
